# Attribution of Runoff Variation in the Headwaters of the Yangtze River Based on the Budyko Hypothesis

**DOI:** 10.3390/ijerph16142506

**Published:** 2019-07-13

**Authors:** Junlong Liu, Jin Chen, Jijun Xu, Yuru Lin, Zhe Yuan, Mingyuan Zhou

**Affiliations:** Changjiang Scientific Research Institute of Changjiang Water Resources Commission, Wuhan 430010, China

**Keywords:** climate change, human activities, runoff changes, headwaters of the Yangtze River

## Abstract

Quantifying the contributions of climate change and human activities on runoff changes is of great importance for water resource management, sustainable water resource utilization, and sustainable development of society. In this study, hydrological and climatic data from hydrological and meteorological stations in the headwaters of the Yangtze River (YRHA) from 1966 to 2013 were used to quantitatively attribute the runoff change to the impacts of climate change and human activities separately. Firstly, the change trends in precipitation, runoff depth and potential evapotranspiration were analyzed by the Mann-Kendall test method. Three methods, secondly, including ordered clustering, Mann-Kendall and cumulative anomaly curve were adopted to detect the change points of runoff at Zhimenda hydrological station and partition the whole study period into two sub-periods at the change point (base and impacted periods). Then, the elasticity coefficient method based on the Budyko hypothesis was applied to calculate elasticity coefficients of runoff to precipitation, potential evapotranspiration and land use/cover during the two periods, and to evaluate the contributions of climate change and human activities. Results indicated that during 1966–2013, runoff depth, precipitation and potential evapotranspiration all showed a significant increasing trend, with increasing rates of 7.26 mm decade^−1^, 18.725 mm decade^−1^ and 7.228 mm decade^−1^, respectively. One change point (2004) was detected for the annual runoff, and 1966–2003 and 2004–2013 were respectively identified as base and impacted periods. The results of elasticity coefficients showed that the runoff depth was most sensitive to the change of precipitation during the two periods. The relative contributions of precipitation, potential evapotranspiration and parameter *n* to runoff changes were 99.7%, −6.08% and 3.88%, respectively. Furthermore, the coupled contribution rate of other factors was less than 2.5%. Generally, results indicated that precipitation is the main factor on the historical runoff changes in this basin.

## 1. Introduction

In recent years, the study of water cycle and water resources evolution in a basin under changing environment has already become a hot topic in the world [1,2]. Climate change and human activities are important components of the changing environment [3], and their impacts are often comprehensive. Generally, climate change affects the hydrological cycles [4] by affecting meteorological factors such as precipitation, potential evapotranspiration and the increase in the occurrence of some extreme hydrometeorological phenomena, etc. [5,6]. At the same time, the impact of human activities on hydrological processes is mainly through land use, soil and water conservation and water conservancy projects to change the underlying surface properties of the basin, so as to lead to spatial-temporal variations in the hydrological cycles and exert impact on runoff [7,8]. As a result, how to accurately separate the comprehensive impacts and quantitatively assess the impacts of climate change and human activities on runoff is one of the key issues of scientific research in hydrology and meteorology [9], which is of great significance for better and clearer ascertaining of the causes of runoff change and for water resources planning and management in a basin [10]. Various statistical methods and hydrological models have been used by numerous scholars to study the effects of climate change and human activities on runoff changes in river basins of China and other countries [11,12,13,14]. However, the limitations of these methods and models are obvious. Studies based on statistical methods not only need long-term records of hydrological and meteorological datasets [15], but also lack a physical basis [16]. As for hydrological models, on the one hand, because hydrological models require enormous input datasets (e.g., surface condition, water intake engineering, and engineering scheduling) to calibrate and validate in the process of modeling, these data may not be available in many cases, so it is difficult to establish accurate hydrological models [15,17]. Therefore, generally speaking, hydrological models are just suitable for the analysis of typical catchments and regions, but they are difficult to popularize and apply in large-scale basins [10,18]. On the other hand, due to the fact that the dominant factors of water cycle processes at different time and watershed scales are significantly different, hydrological models are usually calibrated and validated on small time (monthly and daily scales, etc.) and sub-basins scales. When the results are directly applied to the study of large time scale (year or years) and the whole basin, it still remains to be discussed whether the model and parameters can reflect the dominant process of water cycle and its parameter characteristics on large time scale and the whole basin.

In view of the fact that these limitations exist between statistical methods and hydrological models, the elasticity coefficient method based on the Budyko hypothesis was adopted to quantitatively evaluate the effects of climate change and human activities to the runoff change in this study. Recently, this method has been widely applied in different regions of the world [19,20] for studying the runoff change response to climate change and human activities. Because it not only has a solid scientific base [16], but also is relatively simple: only some meteorological data need to be input and only one parameter needs to be calibrated [10]. Furthermore, the performance of these methods used in large scale catchments can meet the requirements of calculation accuracy and even can perform as good as the more complex hydrological models and statistical analysis [19,21]. However, the method is based on the principle of water balance and energy balance in the basin for many years, and there may be some uncertainty.

The headwaters of the Yangtze River (YRHA) is the starting point of the hydrological cycle throughout the Yangtze River Basin, and is the start-up area of the Yangtze River runoff change, and the early-warning and sensitive area of climate change [22]. It plays a very important role in ensuring the long-term flow of the Yangtze River, and transporting high-quality water resources downstream. In the past 50 years, in the context of global warming, the ecological environment of the YRHA has been deteriorating due to the influence from natural factors together with human activities. Environmental problems such as glacial retreat, wetland shrinkage, grassland degradation and soil erosion have become increasingly prominent [23], which poses a great threat to the water resources and ecological security of the whole Yangtze River Basin. Therefore, the impact of climate change and human activities (e.g., overgrazing, tourism, adventure activities, and construction of the Sanjiangyuan National Nature Reserve, etc) on water resources and ecological environment in the YRHA has recently drawn considerate concerns [22]. However, at present, there are relatively few studies on the systematically quantitative assessment of the effects of climate change and human activities on runoff change in the YRHA according to the related studies reported in literature. In view of the above reasons, this research did a case study in the YRHA by using the elasticity coefficient method to better understand the factors that lead to changes in water resources.

The main objectives of this study are (1) to analyze the trends in runoff depth, precipitation and potential evapotranspiration in the YRHA from 1966 to 2013, (2) to accurately identify change points of runoff for the period of 1966–2013, (3) to assess the elasticity coefficients of runoff to climatic factors and underlying surface conditions, and (4) to quantify the contributions of climate change and human activities on runoff change and discuss the causes of runoff change. The findings of this study can serve as a reference for scientific management of water resource and sustainable development of ecological environment in the YRHA under the influence of climate change and human activities in the future. Meantime, this study can also provide reference for related research on runoff change attribution analysis in other similar basins.

## 2. Study Area and Data

### 2.1. Study Area

**Figure 1:**
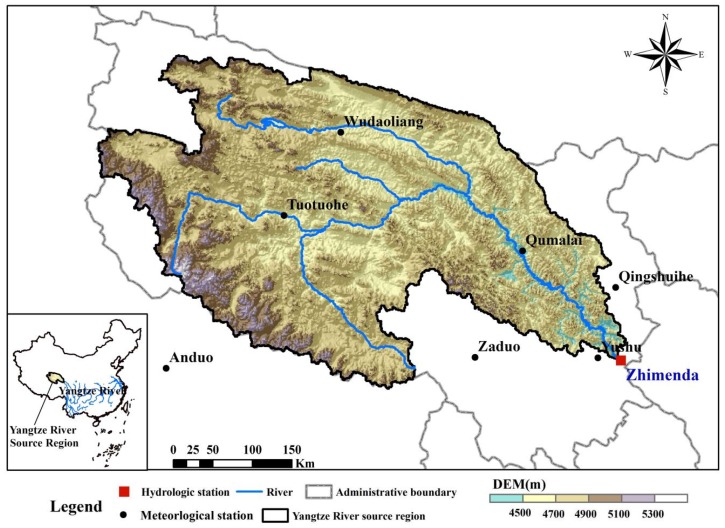
Topographical map of the headwaters area of the Yangtze River.

The headwaters area of the Yangtze River (YRHA, 90°30′ to 97°10′E, 32°30′ to 35°50′N) is located in the southern part of Qinghai Province, China, and in the hinterland of the Qinghai–Tibet Plateau. The drainage area above the Zhimenda hydrological station located at the basin outlet approximately covers an area of 15.7 × 10^4^ km^2^, accounting for 43.6% of the total Three-River Headwaters Region [24] (the Yangtze River, Yellow River and Lantsang River). The peaks around the YRHA are generally above 5500 m, the elevations of other areas are mostly above 4000 m, the average elevation of the YRHA is about 4777 m [25]. The average slope of the basin above Zhimenda hydrological station is about 3.5° [26] (Figure 1). The headwaters area of the Yangtze River belongs to a semi-arid area, where the spatial distribution of precipitation is uneven and gradually decreases from the southeast to the northwest, and is the region with the least precipitation in the Yangtze River basin, with an average annual precipitation of 398 mm. It is located in the sub-frigid zone of the continental plateau and the plateau cold zone, and the average annual temperature of the source area ranges from −5.3 °C to 3.3 °C, with little seasonal variation. The sunshine time is long, and the solar radiation is strong. Frozen soil is widely distributed and basically covers the whole area [11] and glacier area accounts for more than 89% of the whole source area of the Three-River Headwaters Region. Precipitation, glacial meltwater and ice meltwater of frozen soil are the main source of surface water resources in the YRHA. The average annual flow and the average annual surface water resource in the basin are about 408 m^3^/s and 1.29 × 10^10^ m^3^ [24,27], respectively. In August 2000, China officially established the Sanjiangyuan National Nature Reserve in Qinghai province, and the YRHA is an important part of it [11].

### 2.2. Data

The observed daily discharge data used from 1966 to 2013 in this study were obtained from the Zhimenda hydrological station located at the basin outlet (Figure 1). In this research, daily meteorological data during 1966–2013 from seven meteorological stations, three of which are located in the YRHA and four around it (Figure 1), were collected from China Meteorological Data Sharing Service System (http://data.cma.gov.cn). These daily meteorological data consists of daily precipitation, daily average temperature, daily maximum temperature and minimum temperature, sunshine hours, wind speed, etc. The potential evapotranspiration values of seven meteorological stations were calculated according to the Penman-Monteith method [28]. Daily precipitation and potential evapotranspiration data of seven meteorological stations were reorganized into annual precipitation and evapotranspiration. The CoKriging interpretation method [29] had be used to convert annual precipitation and potential evapotranspiration of meteorological stations into the average values of corresponding variables of the basin.

## 3. Methodologies

### 3.1. Trend Test and Change Point Detection

#### 3.1.1. Non-Parametric Mann-Kendall Trend Analysis

In the present study, in order to detect the change trends of precipitation, runoff depth and potential evapotranspiration, the non-parametric Mann-Kendall (MK) test method [30,31] recommended by the World Meteorological Organization was used which has been widely used for trend detection in hydrological and meteorological variables, because it does not need the data to follow any particular distribution and is relatively simple to use. The MK test method is described as follows:

(1) $$S=\sum_{j=1}^{n-1}\sum_{i=j+1}^{n}\mathrm{sgn}(x_{i}-x_{j})$$

in which

(2) $$\mathrm{sgn}(x_{i}-x_{j})=\left\{ \begin{array}{r} 1\;x_{i}>x_{j}\\ 0\;x_{i}=x_{j}\\ -1\;x_{i}<x_{j} \end{array}\right.$$

in which

(3) Var(S)=n(n−1)(2n+5)/18

(4) $$Z=\left\{ \begin{array}{c} (S-1)/\sqrt{Var(S)}\;S>0\\ 0\;S=0\\ (S+1)/\sqrt{Var(S)}\;S<0 \end{array}\right.$$

where *x_j_* and *x_i_* donate the sequential data values, *n* is the length of the dataset. For the two side tests, the null hypothesis is accepted if $\left|Z\right|\leq Z_{\left(1-\alpha /2\right)}$ at *α* level of significance, thus the trend is not significant. The positive and negative values of *Z* represent upward and downward trend, respectively. When the absolute value of *Z* greater than or equal 2.58, 1.96 and 1.28 indicates that it is respectively adopted by the 99%, 95% and 90% confidence level.

Furthermore, the self-correlated of the raw data will, to a certain extent, enhance or reduce the detection ability of MK to trend results [32]. Therefore, Durbin-Watson (DW) is used to detect the self-correlated of hydrometeorological data in this study, the detailed theory of which have been widely described by many previous literatures [32,33].

#### 3.1.2. Change Point Analysis Methods

Identifying change points is of great importance for runoff change analysis [34,35], but change point detection generally has large uncertainty [36]. Therefore in the study, we used multiple analysis methods, namely ordered clustering (OC), MK [37] and cumulative anomaly curve (CAC), to identify the change points. The OC can explore possible change points in the hydrological series by minimizing the sum of the squares of the dispersions between the classes and the relatively large squared deviation between the classes [38]. The CAC is a commonly used method for visually determining the trend of climate change from curves. The cumulative anomaly curve can be drawn from the cumulative anomaly value of hydrological series, and the approximate time of change point can be judged from the obvious fluctuation of the curve [39,40]. After finding the change points, the whole study period (1966–2013) was divided into two sub-periods: the base period (assumed to be natural condition without human activities) and impacted period.

### 3.2. Runoff Change Attribution Identification

#### 3.2.1. Water Balance Model Based on Choudhury-Yang Equation

Hydrological elements for a basin over a long-term timescale follow the water balance principle, and the simplified form of the water balance equation can be expressed as:

(5) R=P−E+ΔS

where *R* is mean annual runoff, *P* is the mean annual precipitation, *E* represents the mean annual actual evapotranspiration, Δ*S* is water storage change in a basin, which approaches zero at a long period of time.

At the catchment scale, runoff depth and precipitation can be obtained from actual observations. The actual evapotranspiration can be calculated by water-energy balance equation pioneered by Budyko [41]. There are various forms of solutions to the Budyko hypothesis, and some representative ones are listed in Table 1.

Among these forms, one of the more common forms is the Choudhury-Yang equation [47,49] based on the Budyko hypothesis and mathematical derivation, which is as follows:

(6) $$E=\frac{E_{0}P}{{\left(P^{n}+{E_{0}}^{n}\right)}^{1/n}}$$

where *E*_0_ is the mean annual potential evapotranspiration, the parameter *n* reflects the underlying surface characteristics, which is mainly related to topography, properties of soil and vegetation cover, etc [49]. Therefore, in this paper, we assume that the change in parameter *n* during the study periods is mainly caused by human activities, and a higher (lower) value of *n* usually corresponds to the lower (higher) land cover and to the higher (lower) land use. Substituting Equation (6) into Equation (5), catchment water balance Equation (5) can be rewritten as:

(7) $$R=P-\frac{E_{0}P}{{\left(P^{n}+{E_{0}}^{n}\right)}^{1/n}}$$

where *R* is annual runoff depth, other parameters are the same as above.

In general, there is no significant changing trend in the catchment characteristics parameter *n* at short time [34]. Therefore in this research, the parameter *n* can be calibrated by Equation (7) using the values of runoff depth, precipitation and potential evapotranspiration from 1966 to 2013.

#### 3.2.2. Elasticity Coefficient

Schaake (1990) [50] defined the precipitation elasticity of runoff as the ratio of the runoff variation rate to the variation rate of precipitation. Similarly, we can have the following form:

(8) $$\varepsilon_{x}=\frac{\partial R/R}{\partial x/x}$$

where *ε_x_* is the elasticity coefficient of the runoff to independent variables, *x* represents *P*, *E*_0_ and *n*, respectively.

In which the elasticities of runoff can be given as:

(9) $$\varepsilon_{P}=\frac{{\left(1+\varphi^{n}\right)}^{1/n+1}-\varphi^{n+1}}{\left(1+\varphi^{n})[{\left(1+\varphi^{n}\right)}^{1/n}-\varphi \right]}$$

(10) $$\varepsilon_{E0}=\frac{1}{\left(1+\varphi^{n})[1-{\left(1+\varphi^{-n}\right)}^{1/n}\right]}$$

(11) $$\varepsilon_{n}=\frac{\ln(1+\varphi^{n})+\varphi^{n}\ln(1+\varphi^{-n})}{n(1+\varphi^{n})[1-{\left(1+\varphi^{-n}\right)}^{1/n}]}$$

where

(12) $$\varphi =\frac{E_{0}}{P}$$

#### 3.2.3. Quantification of Contributions of Climatic Variables and Underlying Surface Changes to Runoff Change

Based on the change points detected, the change of runoff depth, precipitation and underlying surface (*n*) from base period to impacted period can be written as:

(13) $$dx=x_{2}-x_{1}$$

where *dx* is the amount of change from base period to impacted period, *x* represents each of the above three variables, respectively, *x*_1_ and *x*_2_ are the mean annual values of *P*, *E*_0_ and *n* in base period and impacted period, respectively.

Assuming *P*, *E*_0_ and *n* in Equation (7) are independent variables, Equation (7) can be expressed as *R* = *f* (*P*, *E*_0_, *n*). Then, we can get the total differential of *R*, which can be written as:

(14) $$dR^{\prime }=\frac{\partial f}{\partial P}dP+\frac{\partial f}{\partial E_{0}}dE_{0}+\frac{\partial f}{\partial n}dn$$

Then, the contribution of climate change (*P*, *E*_0_) and underlying surface (*n*) to runoff change can be computed following the equation (15).

(15) $$\eta_{x}=\frac{dR_{x}}{dR}\times 100\%$$

where *dR’* is the total value of runoff change caused by *P*, *E*_0_ and *n*, *dR_x_* is runoff change value caused by *P*, *E*_0_ and *n*, respectively, *η_x_* is the contribution of runoff change in *P*, *E*_0_, *n* and coupled other factors (*η_o_*), respectively.

Relative error (RE) was adopted to quantify the performance of the elasticity coefficient method used in this study, which was defined as follows:

(16) $$RE=\frac{R_{sim}-R_{obs}}{R_{obs}}\times 100\%$$

where *R_sim_* and *R_obs_* are the values of the simulated and observed runoff depth, respectively.

## 4. Results

### 4.1. Trend Analysis of Hydrometeorological Variables

**Figure 2:**
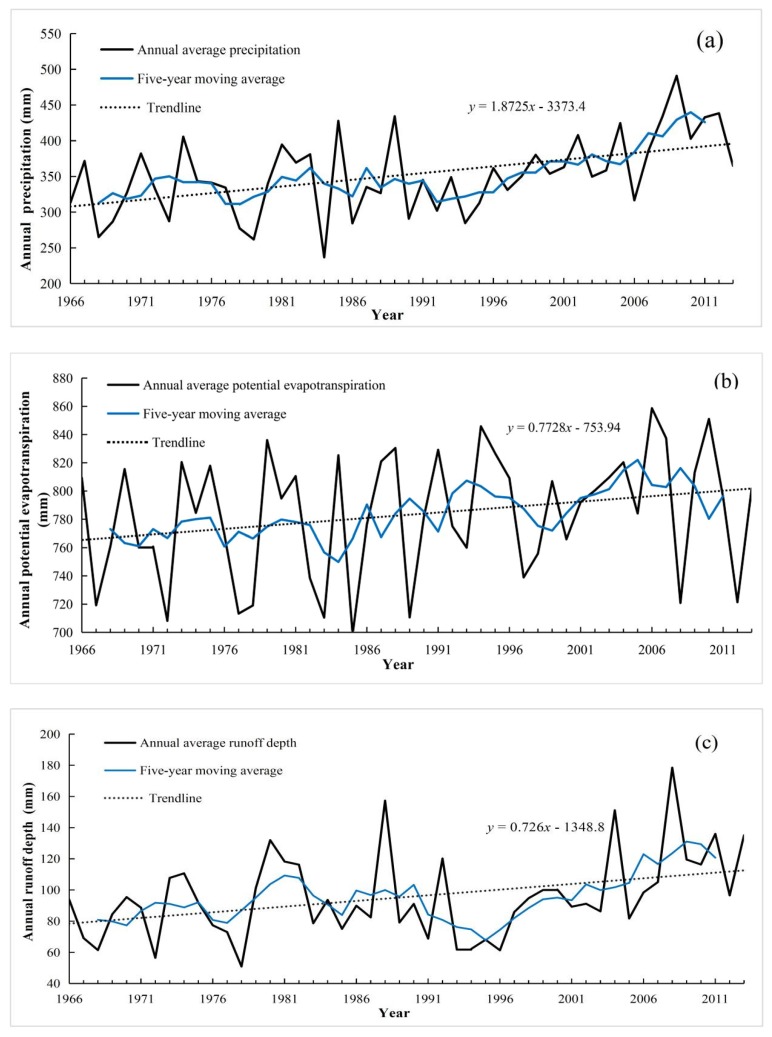
The change trend of annual precipitation (**a**), potential evapotranspiration (**b**) and runoff depth (**c**) from 1966 to 2013 in the YRHA.

The analysis of hydrometeorological elements is important for the preliminary recognition of runoff changes, and is also the basis of this study, which can reflect the change of runoff from the side and can verify the accuracy of runoff analysis results. The changes of precipitation, potential evapotranspiration and runoff depth from 1966 to 2013 are illustrated in Figure 2 and the statistical characteristics are listed in Table 2. It can be concluded that precipitation, potential evapotranspiration and runoff depth were independent (that is to say, they were not self-correlated), and they all showed a fluctuant increasing trend from 1966 to 2013, with increasing rates of 18.725 mm decade^−1^, 7.228 mm decade^−1^ and 7.26 mm decade^−1^, respectively. When compared with them, it can be found that the increasing trend of precipitation was more significant. At the same time, average annual values of precipitation, potential evapotranspiration and runoff depth showed averages of 351.92 mm, 783.60 mm and 95.53 mm, respectively, for the 48-year study period. What is more, the results of MK trend test show that the test statistics of runoff depth and precipitation both were very significant, over the past 48 years, reaching 95% and 99% confidence levels, respectively. The potential evapotranspiration reached a 90% confidence level, indicating test statistic was significant.

### 4.2. Attribution of Runoff Variation

#### 4.2.1. Change Point Analysis of Runoff

**Figure 3:**
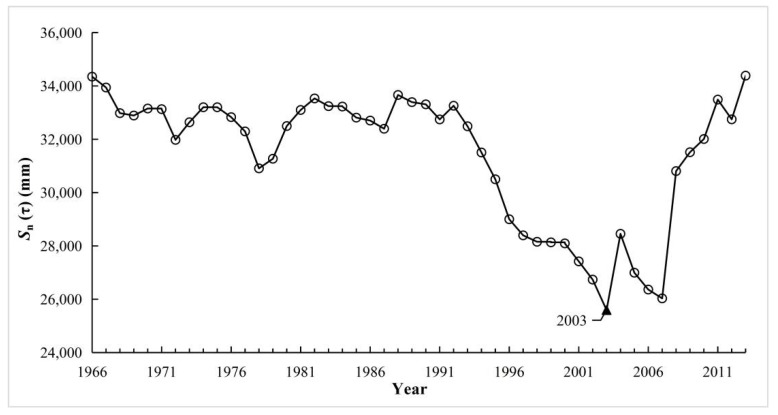
*S_n_*(*τ*) change curve of annual runoff depth by ordered clustering (OC) analysis method from 1966 to 2013 in the YRHA.

**Figure 4:**
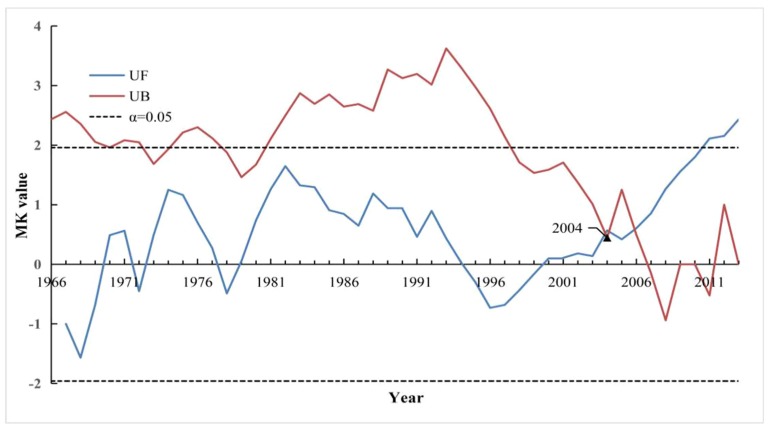
Mann-Kendall (MK) test of annual runoff depth from 1966 to 2013 in the YRHA.

**Figure 5:**
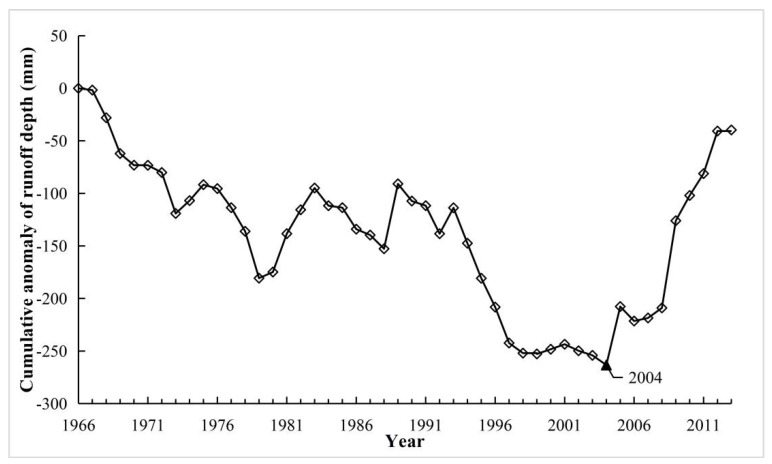
Cumulative anomaly curve (CAC) of annual runoff depth from 1966 to 2013 in the YRHA.

We used 3 methods (the OC, MK and CAC methods) to detect change point of runoff over the study period of 1966–2013, as shown in Figure 3, Figure 4 and Figure 5. As can be seen, for the OC analysis method, the change point for runoff occurred in 2003, but the same change point detected by MK test (confidence level α = 0.05, the critical value for the normal distribution U_α/2_ = ± 1.96) and CAC methods was 2004. Therefore, integrating the results of the above three methods, it can be concluded that 2004 is identified as the change point of runoff in this study, and this conclusion also was identical to the results found in a previous related literature carried by Li (2018) [3]. Consequently, the base period in the YRHA can be determined to be 1966–2003, and 2004–2013 was taken as impacted period.

#### 4.2.2. Sensitivity Analysis of Runoff to Climatic Variables and Underlying Surface Changes

The elasticity coefficients of the runoff to the precipitation, potential evapotranspiration and parameter *n* during base and impacted periods in the YRHA, as well as the changes of grassland and land cover in the two periods are shown in Table 3. Compared with the base period, the average annual potential evapotranspiration, runoff depth and precipitation period presented increasing trends in the impacted period, with relative changes of 2.72%, 37.53% and 19.85%, respectively. In contrast, the parameter *n* reflecting the underlying surface characteristics exhibited a slight decreasing trend, decreasing from 1.10 in the base period to 1.09 in the impacted period. It may be mainly caused by changes in grassland and bare land cover in recent years, the impact of which on runoff is mainly reflected in the impact on evapotranspiration. What is more, the drought index (*E_0_/P*) decreased by −14.72% compared with the base period, indicating that the climate in impacted period was warmer and wetter than that in the base period in the YRHA.

In general, runoff is negatively correlated with potential evapotranspiration and parameter *n*, but positively correlated with precipitation. The calculated elasticity coefficients implied that in the base period, when potential evapotranspiration or parameter *n* increases by 10%, runoff depth would decrease by 8% or 15.3 %, respectively, and a 10% precipitation increase would result in a 18% increase in runoff depth. In the impacted period, when potential evapotranspiration or parameter *n* increases by 10 %, runoff depth would decrease by 7.5% or 13.4 %, respectively, and a 10 % increase in precipitation would increase runoff depth by 17.5%. In addition, the largest of the absolute values of the elasticity coefficient was precipitation, followed by parameter *n*, and the smallest was potential evapotranspiration, indicating that the runoff depth was highest sensitive to the change of precipitation. From the absolute values of the three calculated elasticity coefficients between the two periods, the sensitivity of runoff depth to them was slightly reduced to a certain extent, suggesting that runoff depth was increasingly sensitive to changes in other factors which have not carefully considered in this study.

#### 4.2.3. Attribution Identification of Runoff Change

The contributions of precipitation, potential evapotranspiration and parameter *n* to runoff depth change were calculated according to equation (15) with the results shown in Table 4. As can be seen from Table 4, the difference between the calculated runoff depth change (*dR′*) by the elasticity coefficient method and the actual runoff depth change (*dR*) was very small, with the difference only being −0.84, and the value of relative error was only −2.5%. Therefore, the findings can illustrate that the elasticity coefficient method used in this study is comparatively accurate in quantitatively assessing the response of runoff change to climate change (precipitation, potential evapotranspiration) and human activities (parameter *n*) in the headwaters of the Yangtze River. Within the total amount of change, the increment caused by precipitation change is approximately 33.14 mm and the contribution rate of precipitation change to runoff depth change is 99.7 %. The increment caused by parameter *n* change is approximately 1.29 mm, with the contribution rate of parameter *n* change to depth runoff change being 3.88 %. However, the reduction caused by potential evapotranspiration is approximately 2.02 mm, whilst the contribution rate of potential evapotranspiration change to depth runoff change is −6.08 %. Moreover, the coupled contribution rate of other factors which were not currently involved in the specific analysis and the errors made from Equations (14) and (15) are 2.5% in this study. Consequently, these results indicate that the impact of precipitation is the main factor causing the runoff change in the YRHA from 1966 to 2013, followed by the change of the potential evapotranspiration and parameter *n.*

## 5. Discussion

### 5.1. Factors Affecting the Accuracy of the Assessment Results

Human direct access to water can greatly affect the accuracy of calculation. However, due to the fact that there are no large water conservancy projects (e.g., large and medium-sized reservoirs, large-scale agricultural irrigation areas, etc) in the YRHA for the time being, therefore, the impact of human direct access to water on runoff can be temporarily ignored in this study. What is more, scarce hydrological station data may also limit the simulation accuracy. At present, due to the relative lack of hydrological stations with long series of hydrological observation data in the YRHA, only one hydrological data of the control station was used in this study. Therefore, the spatial representativeness of hydrological data and the accuracy of this study may still have some limitations, which may need to wait for more observation data to solve in future.

### 5.2. Impacts of Glaciers and Frozen Soil on the Runoff Change

The Qinghai-Tibet Plateau is the most developed area of frozen soil and glaciers in China, the hinterland of which is the YRHA. However, in recent years, the trend of warm and wet weather on the Qinghai-Tibet Plateau has been remarkable in the context of global warming and its associated climate. Hence, the impact of frozen soil and glaciers on runoff change cannot be ignored more and more. As far as parameter *n* is concerned, in this paper, we only showed that the decrease of parameter *n* may be related to grassland and bare land cover in recent years. However, as the temperature in the YRHA increases, the frozen soil is gradually melting, which changes the energy transmission and material migration in the near earth and the soil moisture condition of the active layer (water holding capacity, water conductivity, etc.) [51,52]. Therefore, the decrease in the parameter *n* may have a certain relationship with the melting of frozen soil. But in view of the complexity of the mechanism that temperature can affect underlying surface and runoff process by influencing the melting of frozen soil and the weak foundation of relevant research in this region, it cannot be explained accurately in this study and more detailed research is also needed to carry out in the future. Furthermore, the effect of temperature on runoff in the YRHA increases is not consistent with that in the wet regions of eastern China, which is mainly reflected in that it can affect the melting of glaciers and frozen soil of the YRHA. Although the paper gives the conclusion that the sensitivity of runoff to other factors has been increased and gives the coupled contribution rate of other factors, the contribution of meltwater of glaciers and frozen soil to runoff change has not been accurately given yet due to the unavailable data, so relevant results and conclusions also need to be further calculated and validated by collecting relevant data in subsequent studies.

### 5.3. Several Issues to be Further Studied

Although the effects of climate (precipitation, potential evapotranspiration) and human activities (parameter *n*) on runoff have been quantitatively obtained based on the elasticity coefficient method in this paper, it should be noted that there are still some issues that need to deserve further study. In this study, the impact of human direct access to water on runoff was temporarily ignored due to the absence of large-scale water conservancy projects in the study area, still this does not mean that this kind of impact should be totally ignored. Generally, the impact of human activities on runoff is usually integrated [53], including both land use and cover changes, as well as the direct impact of direct access to water. In this study, human activities were regarded as a whole parameter *n*, but how to further refine the impact of different human activities on runoff is a challenge and needs to be further analyzed in studies conducted in future. Traditionally, most of these kinds of studies on the impacts of climate change and human activities on runoff mainly focused on different catchment scenarios to carry out. In fact, the melting of glaciers and frozen soil mainly occurs in relatively high temperature seasons (e.g., summer). Hence, whether the contribution rate of glaciers and frozen soil to runoff change can be studied on a different monthly or seasonal scales is also a problem worthy of further study, which can provide new ideas for further study of the impact of runoff change in the YRHA or other similar watersheds in the world. Furthermore, the conclusion of this paper was that precipitation was the dominant factor of runoff change. As the sentinel of the impact of climate change [54], the hydrological process of the YRHA was very sensitive to climate change. Therefore, our next research will predict potential effects of future climate change on runoff in the YRHA based on relevant data and methods, which will contribute to the basin-wide adaptive management to some extent in a changing environment.

## 6. Conclusions

In this paper, the YRHA was selected as the study area. The change trends in precipitation, runoff depth and potential evapotranspiration during 1966–2013 were analyzed by using the MK test method. At the same time, we used three methods (the OC, MK and CAC methods) to detect change point of runoff over the past 48 years. Then, the impacts of climate change (precipitation, potential evapotranspiration) and human activities (parameter *n*) on runoff were quantified by the elasticity coefficient method. The main conclusions are presented as follows:

(1) During the period from 1966 to 2013, runoff depth, precipitation and potential evapotranspiration all showed a significant increasing trend in the headwaters of the Yangtze River, with increasing rates of 7.26 mm decade^−1^, 18.725 mm decade^−1^ and 7.228 mm decade^−1^, respectively.

(2) One change point detected in this study happened in 2004. Hence, based on this conclusion, 1966–2003 and 2004–2013 were respectively identified as base and impacted periods of the YRHA.

(3) According to the results of elasticity coefficients calculated in this study, it can be concluded that runoff depth was most sensitive to the change of precipitation, followed by parameter *n*, and finally potential evapotranspiration, whether in the base or the impacted periods. The relative contributions of climate change (precipitation, potential evapotranspiration) and human activities (parameter *n*) to runoff changes were 99.7%, −6.08% and 3.88%, respectively. At the same time, the coupled contribution rate of other factors which were not currently involved in the specific analysis was less than 2.5%. In general, results indicate that the impact of precipitation is the main factor on the runoff changes in the YRHA.

## Figures and Tables

**Table 1:** Different forms of solutions to the Budyko hypothesis.

Formula	Parameter	Reference
$E=P[1-\exp(-E_{0}/P)]$	none	Schreiber (1904) [42]
$E=E_{0}\tan\;h(P/E_{0})$	none	Ol’dekop (1911) [43]
$E=P/{\left[1+{\left(P/E_{0}\right)}^{2}\right]}^{0.5}$	none	Pike (1964) [44]
$\begin{array}{l} E=\{P[1-\exp(-E_{0}/P)]\cdot \\ E_{0}\tanh(P/E_{0}){\}}^{0.5} \end{array}$	none	Budyko (1958) [45]
$E=P{\left[1+\varphi -(1+\varphi^{\omega })\right]}^{1/\omega }$	*ω*	Fu (1981) [46]
$E=P/{\left[1+{\left(P/E_{0}\right)}^{\alpha }\right]}^{1/\alpha }$	*α*	Choudhury (1999) [47], Yang et al. (2008) [48]

**Table 2:** Results of change characteristics and MK trend test of precipitation, potential evapotranspiration and runoff depth from 1966 to 2013 in the headwaters of the Yangtze River (YRHA).

Variables	Durbin-Watson (DW) Test	Average Annual Value (mm)	Change Ratio (mm/10 a)	Mann-Kendall (MK) Trend Test	
				Test Statistics	Significance Level
*P*	-	351.92	18.725	2.90	0.01
*E*_0_	-	783.60	7.228	1.65	0.10
*R*	-	95.53	7.26	2.43	0.05

Note: when confidence level α = 0.1, 0.05 and 0.01, the critical values of statistical test Z_α/2_ = ± 1.28, ± 1.96 and ± 2.58, respectively; “+” and “−” denote the existence of autocorrelation and non-existence of autocorrelation (α = 0.05), respectively.

**Table 3:** Sensitivity of *R* to *P*, *E*_0_ and *n* during 1966–2003 (base period) and 2004–2013 (Impacted period) in the YRHA.

Period	*E*_0_ (mm)	*R* (mm)	*P* (mm)	*n*	*E*_0_/*P*	Elasticity Coefficient			Area Proportion (%)	
						*ε_E_*_0_	*ε_p_*	*ε_n_*	Grassland	Bare Land
Base period (1966–2003)	779.18	88.60	337.95	1.10	2.31	−0.80	1.80	−1.53	84.66	13.56
Impacted period (2004–2013)	800.40	121.85	405.023	1.09	1.97	−0.75	1.75	−1.34	84.98	13.20
Change (*δ*)	21.22	33.25	67.073	−0.01	−0.34	0.05	−0.05	0.19	0.32	−0.36
Relative change (%)	2.72	37.53	19.85	−0.91	−14.72	−6.25	−2.80	−12.42	0.37	−2.62

Note: change the difference of hydroclimatic variables between base period and impacted period, relative change the ratio between *δ* and the mean value in the base period.

**Table 4:** Contributions of *P*, *E*_0_, *n* and coupled other factors to runoff depth change.

Base Period	Impacted Period	*dR_P_*	*dR_E_*_0_	*dR_n_*	*dR*	*dR*′	Δ	RE (%)	Contribution Ratio			
									*η_P_*	*η_E_*_0_	*η_n_*	*η_o_*
1966–2003	2004–2013	33.14	−2.02	1.29	33.25	32.41	−0.84	−2.5	99.7	−6.08	3.88	2.5

Note: Δ the difference between dR’ and dR.
